# Histological spatial analysis on the induction of PD-L1^+^ macrophages by CD8^+^ T cells at the marginal microenvironment of triple-negative breast cancer

**DOI:** 10.1007/s12282-023-01507-9

**Published:** 2023-10-04

**Authors:** Kazushi Suzuki, Rintaro Ohe, Takanobu Kabasawa, Takumi Kitaoka, Masaaki Kawai, Fuyuhiko Motoi, Mitsuru Futakuchi

**Affiliations:** 1 Department of Pathology, Faculty of Medicine, Yamagata University, 2-2-2 Iida-Nishi, Yamagata, 990-9585 Japan; 2 Department of Surgery 1, Faculty of Medicine, Yamagata University, Yamagata, Japan

**Keywords:** Triple-negative breast cancer (TNBC), Programmed death-ligand 1 (PD-L1), Macrophage, CD8^+^ T cell, Tumor microenvironment

## Abstract

**Background:**

Programmed death-ligand 1 (PD-L1) plays important roles in the evasion of antitumor immunity. Because we observed the localization of PD-L1-positive (PD-L1^+^) cells in the marginal region of triple-negative breast cancer (TNBC) specimens, we hypothesized that the marginal microenvironment of TNBC would involve the induction of PD-L1^+^ cells.

**Methods:**

One hundred and one TNBC surgical specimens were examined. We performed immunohistochemical (IHC) studies of PD-L1, CD68, CD8, and pan-cytokeratin in these specimens. We analyzed the localization of IHC-positive cells and the distance between these cells by histological spatial analysis.

**Results:**

In 30.7% of TNBC specimens, PD-L1^+^ cells were located in the marginal region. Approximately three PD-L1^+^ cells accumulated around a single TNBC cell. Most PD-L1^+^ cells were located within 50 μm of TNBC cells. PD-L1^+^ cells were indicated to interact with TNBC cells in the marginal region. PD-L1^+^CD68^+^ cells were located in the marginal region, while CD68^+^ macrophages (MΦs) were observed either in the marginal region or the core region. PD-L1 expression in MΦs was induced in the marginal region. The colocalization of CD8^+^ T cells in the marginal region indicates that PD-L1 expression in MΦs would be induced by interaction with CD8^+^ T cells. Because CD8^+^ T cells are positive for CCL2, CCL2 may induce PD-L1 expression in MΦs.

**Conclusion:**

At the marginal microenvironment of TNBC, PD-L1 expression would be induced in MΦs by interaction with CD8^+^ T cells through CCL2. The interaction between PD-L1^+^ MΦs and TNBC cells would facilitate the growth of TNBC under antitumor immunity. These interactions would be potential targets for restoring antitumor immunity and suppressing TNBC progression.

**Supplementary Information:**

The online version contains supplementary material available at 10.1007/s12282-023-01507-9.

## Introduction

Triple-negative breast cancers (TNBC) are defined as breast cancers without the expression of estrogen receptor (ER), progesterone receptor (PR), and human epidermal growth factor receptor 2 (HER2) [1]. The frequency of TNBC has been reported to be 16% of all breast cancer patients in non-African American, and 8% in Japanese [2, 3]. Although patients with TNBC have been treated with surgical resection or systemic chemotherapy, the high rates of recurrence and distant metastasis would contribute to poor prognosis of patients [1, 4].

Recently, programmed death-ligand 1 (PD-L1) and its receptor PD-1 have been proposed as good targets for treatment [4, 5]. PD-1/PD-L1 signaling has been shown to play important roles in the evasion of antitumor immunity [6, 7]. PD-1/PD-L1 inhibitors have been used for non-small cell lung cancer [8] and for TNBC [4, 5]. The expression of PD-L1 has been shown to be upregulated in cancer cells. Additionally, the expression of PD-L1 has been shown to be upregulated in stromal cells such as macrophages (MΦs) at the tumor microenvironment of TNBC specimens [9, 10].

The tumor microenvironment has been provided the conditions such as tumor promotion and immune suppression [11]. The microenvironment in the marginal region of the tumor specimens would be markedly different from that in the core region because the dynamic interaction between cancer cells and stromal cells would be elicited in the marginal region [12]. Immune cell-derived cytokines at the tumor microenvironment have been reported to increase the expression of PD-L1 [13, 14].

In the tumor-stromal interaction observed in various cancers, such as colon [15], gastric [16], and breast cancer [17], MΦs have been shown to play important roles in tumor progression. MΦs have been demonstrated to be induced by TNBC cells through the cytokines [18, 19]. The interaction between MΦs and TNBC cells has been demonstrated to be involved in tumor progression [17]. In addition, the interaction between MΦs and CD8^+^ T cells has been demonstrated to prevent antitumor immunity by CD8^+^ T cells [20].

CD8^+^ cytotoxic T cells play major roles in antitumor immunity [7, 21]. CD8^+^ T cells have been shown to induce apoptosis in cancer cells by producing cytotoxic granules such as granzyme and perforin [22]. Antitumor immunity mediated by CD8^+^ T cells has been demonstrated to be suppressed by PD-1/PD-L1 signaling [7, 21]. CD8^+^ T cells have been reported to be localized in the marginal region in approximately 30% of TNBC specimens [23, 24].

In our preliminary study, we observed that PD-L1 was expressed in stromal cells in the marginal region of TNBC specimens. Based on these findings, we hypothesized that the tumor microenvironment in the marginal region of TNBC would be involved in the induction of PD-L1^+^ cells. Here, we examined whether PD-L1^+^ cells accumulating in the marginal region would interact with TNBC cells. We also examined whether CD8^+^ T cells would induce the expression of PD-L1 in MΦs in the marginal region of TNBC specimens by histological spatial analysis. Our results indicate that these interactions would be potential targets for restoring antitumor immunity and suppressing TNBC progression.

## Materials and methods

### Patients and tissue specimens

One hundred and one patients who were treated with surgical resection and were diagnosed with TNBC at Yamagata University Hospital, Yonezawa City Hospital, and Sanyudo Hospital from 2009 to 2019 were included. Excised tissues were fixed in 10% neutral-buffered formalin at room temperature and embedded in paraffin. The clinicopathological information, including age, pathological stage, and BRCA1/2 gene mutations, was retrieved from medical records. Nuclear grade was evaluated on each hematoxylin and eosin-stained slide by two pathologists (K.S. and T.K.). This study was approved by the Research Ethics Committee of Yamagata University Faculty of Medicine (2019-342) and was performed in accordance with the Declaration of Helsinki.

### Immunohistochemical (IHC) studies

All IHC studies were performed on a BOND RX autostainer (Leica Biosystems, Nussloch, Germany) according to BOND’s procedures. Positive reactions were detected as brown coloration with 3,3′-diaminobenzidine (DAB; BOND Polymer Refine Detection, Leica Biosystems). The sections were counterstained with hematoxylin (BOND Polymer Refine Detection, Leica Biosystems). Multiplex immunofluorescence (IF) staining was performed using the tyramide signal amplification-based Opal method with an Opal 4-Color Automation IHC Kit (Akoya Biosciences, Marlborough, MA, USA). Opal reagents (Opal520; FITC, Opal570; TRITC) were used alternatively to DAB IHC reagents. The sections were counterstained with spectral DAPI. Multiplex staining was evaluated with an All-in-One Fluorescence Microscope (BZ-X810, KEYENCE, Osaka, Japan). The following primary antibodies were used for IHC studies: PD-L1 (SP142, Abcam, Cambridge, UK), pan-cytokeratin (AE1/AE3, Abcam), CD68 (514H12, Leica Biosystems and KP1, Dako, Agilent Technologies, Santa Clara, CA, USA), CD8 (4D1, Leica Biosystems), CCL2 (2D8, Novus Biologicals, Littleton, CO, USA), p-STAT3 (D3A4, Cell Signaling Technology, Danvers, MA, USA), and p-IκBα (B-9, Santa Cruz Biotechnology, Dallas, TX, USA). Across 101 TNBC specimens, PD-L1 expression on IHC was evaluated by two pathologists (K.S. and T.K.). Based on the criteria of the IMpassion130 trial [4], we considered the cases in which PD-L1^+^ stromal cells occupied ≥ 1% as positive for stromal cells. Similarly, we considered the cases in which PD-L1^+^ TNBC cells occupied ≥ 1% as positive for TNBC cells.

### Histological spatial analysis

All slides were converted to digital whole slide images by MoticEasyScan (Motic Digital Pathology, San Francisco, CA, USA). Histological spatial analyses were performed by HALO software (Indica Labs, Corrales, NM, USA). HALO detected all IHC-positive cells stained with DAB on each slide, such as PD-L1, CD68, CD8, and pan-cytokeratin. The density of IHC-positive cells was analyzed on each slide and visualized as a heatmap. To demonstrate the accumulation of the cells in the marginal region, the density was calculated in the area from 300 μm inside to 300 μm outside the tumor margin and analyzed as a histogram. In the core region, 1 mm^2^ square areas were randomly chosen. The density was calculated in each area and analyzed as a bar chart. To demonstrate the cell-to-cell interaction, we examined the proximity of a cell to another cell and whether several cells surrounded another single cell. For example, HALO allocated PD-L1^+^ cells and pan-cytokeratin^+^ cells to blue dots and yellow dots, respectively. Blue dots and yellow dots were synchronized in one chart. Then, HALO was used to draw the shortest lines from blue dots to yellow dots and measured the length of the lines. Additionally, the number of PD-L1^+^ cells surrounding a single pan-cytokeratin^+^ cell was counted in randomly chosen square areas in the marginal region.

## Results

### PD-L1 IHC studies in TNBC specimens

**Fig. 1:**
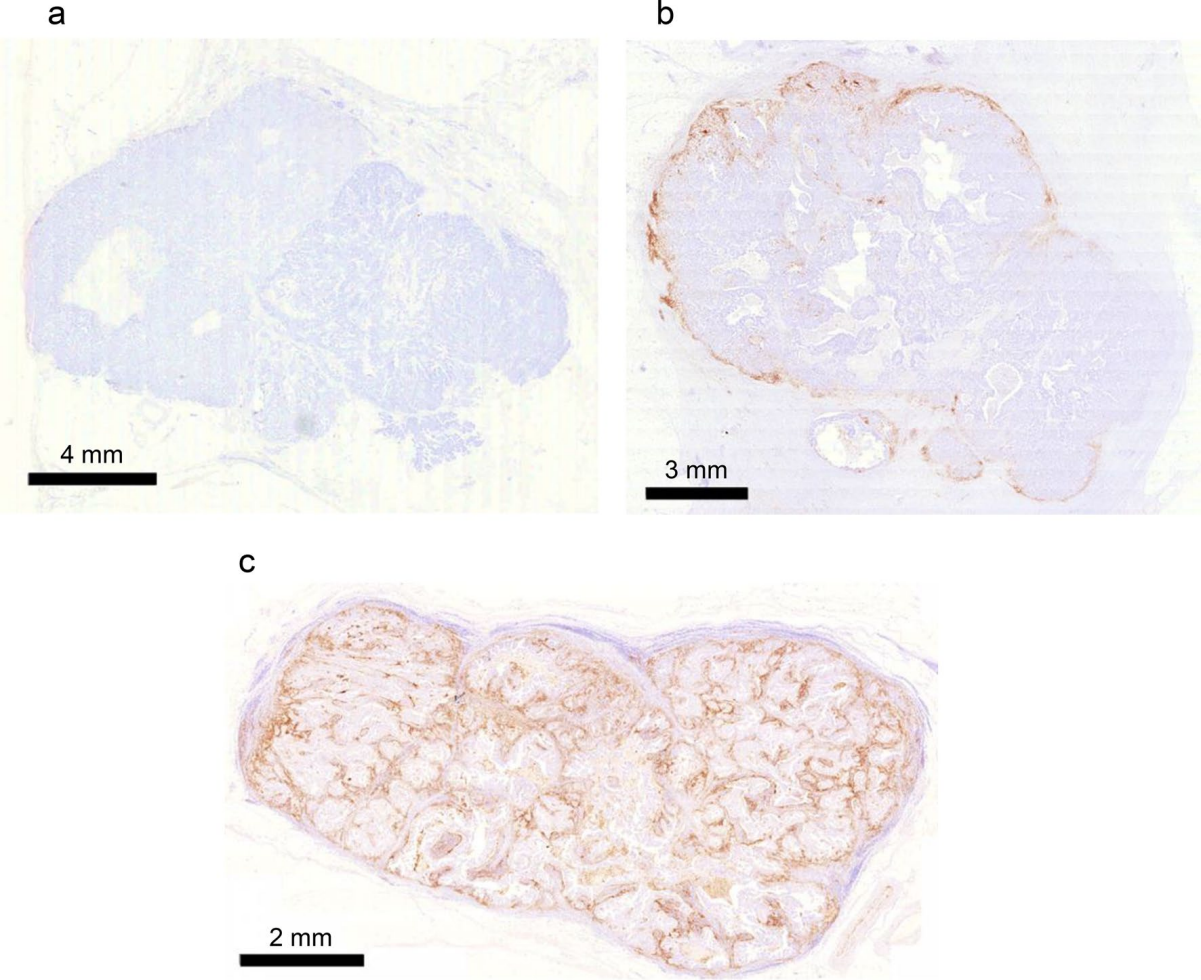
Representative images of PD-L1 IHC studies in TNBC specimens. **a** No PD-L1^+^ cells were observed in 50.5% of TNBC cases. **b** PD-L1^+^ stromal cells and PD-L1^−^TNBC cells were observed in 30.7% of cases. **c** PD-L1^+^ stromal cells and PD-L1^+^ TNBC cells were observed in 18.8% of cases

A summary of clinicopathological characteristics for 101 patients with TNBC was shown in Supplemental Table S1. The available data of BRCA1/2 gene mutations was also shown. To demonstrate the localization of PD-L1^+^ cells in TNBC specimens, we examined specimens with PD-L1 IHC staining. In 50.5% (51/101) of TNBC cases (Fig. 1a), no PD-L1^+^ cells were observed. In 30.7% (31/101), PD-L1^+^ stromal cells and PD-L1-negative (PD-L1^−^) TNBC cells were observed (Fig. 1b). In 18.8% (19/101), PD-L1^+^ stromal cells and PD-L1^+^ TNBC cells were observed (Fig. 1c).

### Accumulation of PD-L1^+^ cells

**Fig. 2:**
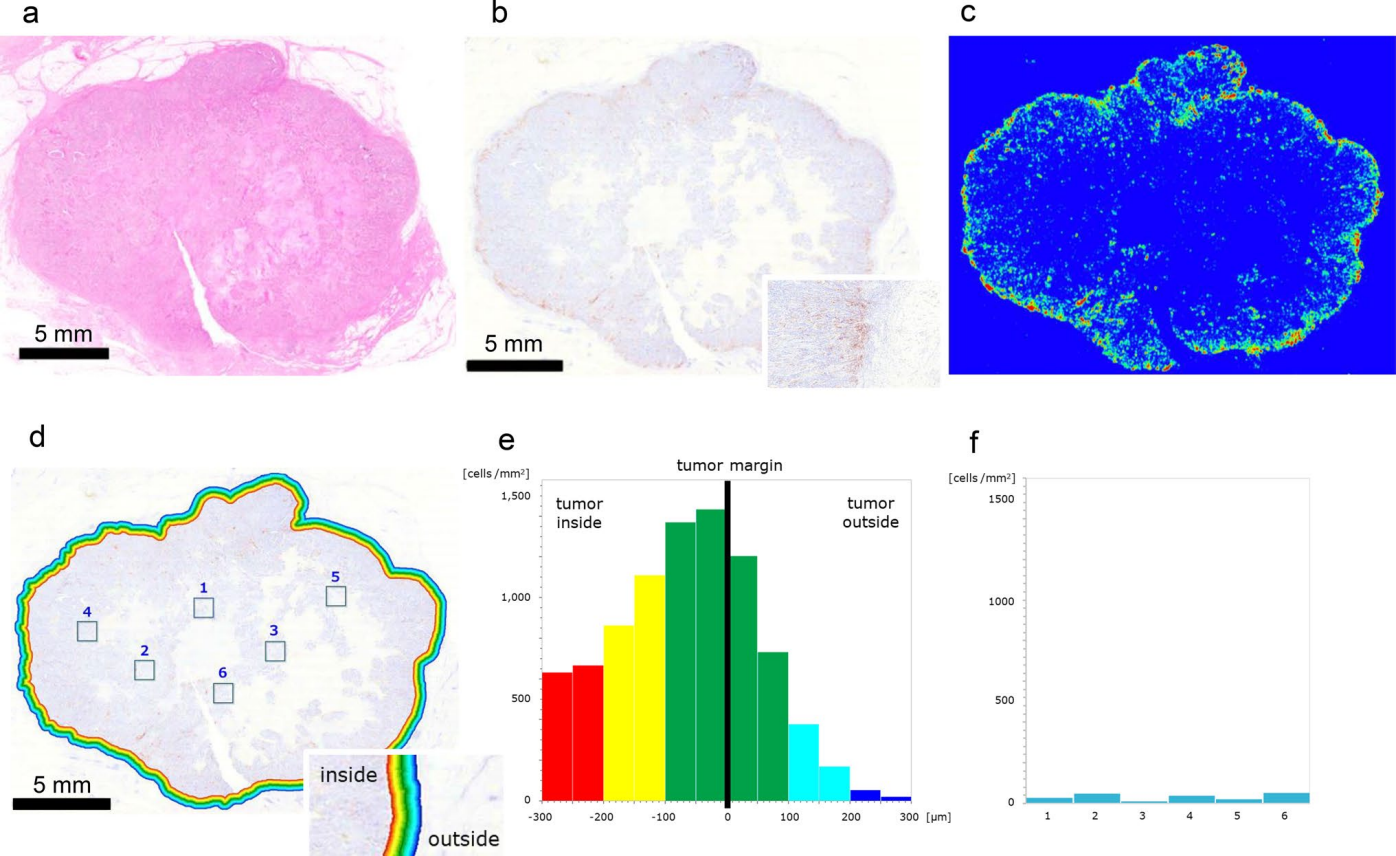
Accumulation of PD-L1^+^ cells. **a** Gross image of a TNBC specimen diagnosed as invasive ductal carcinoma. **b** PD-L1^+^ cells were observed in the marginal region; inset, marginal region. **c** The heatmap image generated by HALO confirmed the accumulation of PD-L1^+^ cells in the marginal region. **d** Quantitative analyses of the density of PD-L1^+^ cells in the marginal region and at the core region; inset, marginal region. **e** The density of PD-L1^+^ cells near the tumor margin. The marginal region was divided into twelve areas. **f** Density of PD-L1^+^ cells in the core region

One TNBC specimen was diagnosed as invasive ductal carcinoma (Fig. 2a). The IHC study of PD-L1 revealed that PD-L1^+^ cells were located in the marginal region (Fig. 2b). The heatmap image generated by HALO revealed the high density of PD-L1^+^ cells in the marginal region (Fig. 2c). The marginal region of TNBC specimen was divided into twelve areas from 300 μm inside to 300 μm outside the tumor margin, and the density of PD-L1^+^ cells was examined in each area (Fig. 2d). Histogram analysis revealed that the density of PD-L1^+^ cells gradually increased toward the tumor margin, and the highest density was 1,435 cells per mm^2^ in the column of – 50–0 μm inside from the tumor margin (Fig. 2e). The density gradually decreased, and the lowest density was 21.2 cells per mm^2^ in the column of 250–300 μm outside from the tumor margin (Fig. 2e). To examine the density of PD-L1^+^ cells in the core region, we randomly chose six square areas (Fig. 2d), and the density of PD-L1^+^ cells in these areas was between 11.0 and 52.0 cells per mm^2^ (Fig. 2f). HALO also revealed the high density of PD-L1^+^ cells in the marginal region of three other TNBC specimens (Supplemental Fig. S1a–i).

### Proximity of PD-L1^+^ cells to TNBC cells

**Fig. 3:**
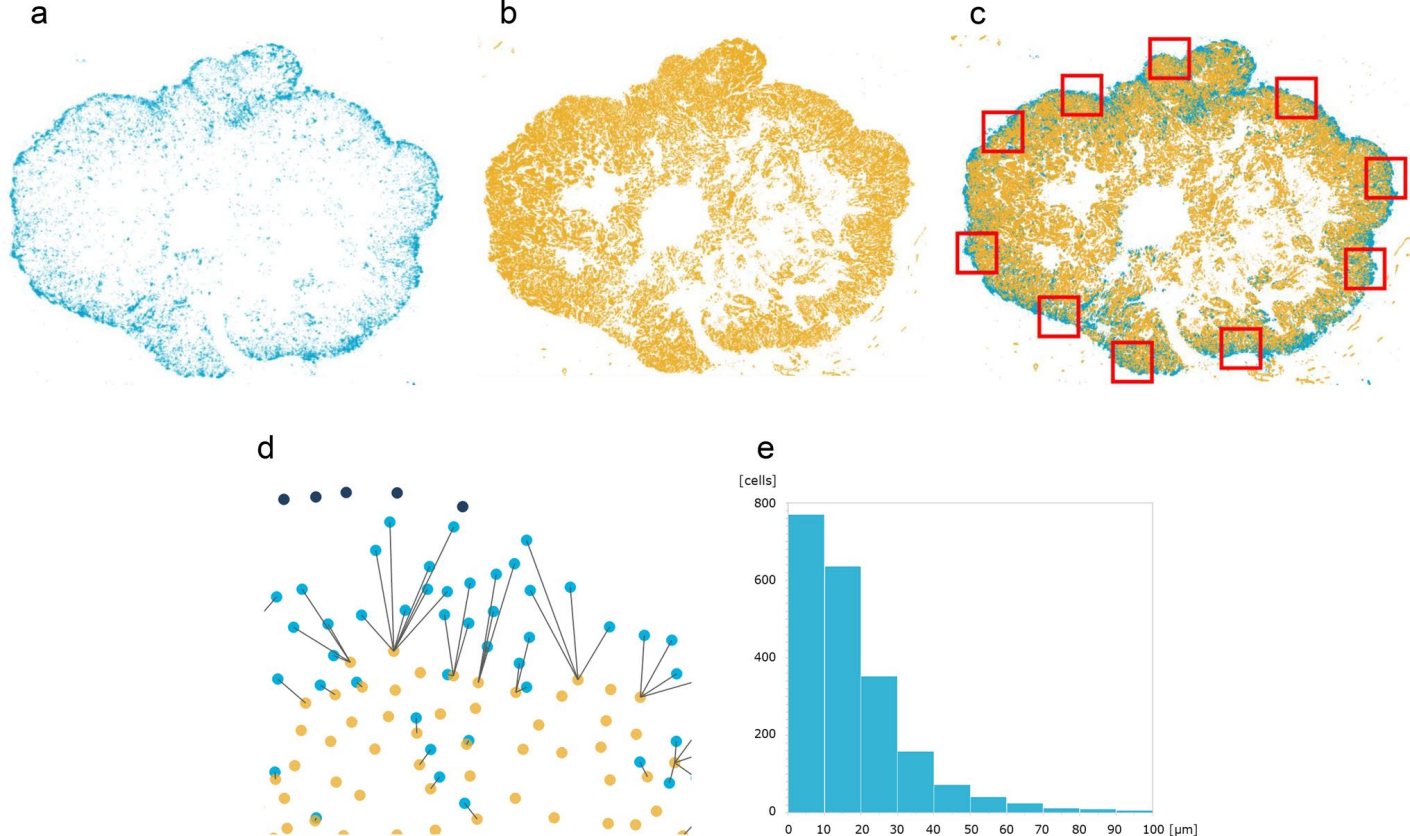
Proximity of PD-L1^+^ cells to TNBC cells. **a** Allocation of PD-L1^+^ cells to light blue dots. Light blue dots were mainly located in the marginal region. **b** Allocation of pan-cytokeratin^+^ TNBC cells to bright yellow dots. Blight-yellow dots were diffusely located in the TNBC specimen. **c** Synchronized chart of A and B. In ten areas in the marginal region (red square), the distance between PD-L1^+^ cells and TNBC cells was examined. **d** Shortest lines from PD-L1^+^ cells to TNBC cells; PD-L1^+^ cells within 50 μm of TNBC cells (light blue dots), PD-L1^+^ cells more than 50 μm away (deep blue dots). **e** Histogram analysis of the distance between PD-L1^+^ cells and TNBC cells. Approximately 95% of PD-L1^+^ cells were located within 50 μm of TNBC cells

HALO allocated PD-L1^+^ cells to light-blue dots in the grid chart (Fig. 3a) and pan-cytokeratin^+^ TNBC cells to bright-yellow dots (Fig. 3b). Light-blue dots and blight-yellow dots were synchronized in one chart (Fig. 3c). We randomly chose ten square areas in the marginal region of TNBC specimen (Fig. 3c). In each square area, HALO was used to draw the shortest lines between PD-L1^+^ cells and TNBC cells (Fig. 3d). PD-L1^+^ cells within 50 μm of TNBC cells were allocated to light blue dots, and cells more than 50 μm away from TNBC cells were allocated to deep blue dots (Fig. 3d). We counted the number of PD-L1^+^ cells per single TNBC cell in these square areas (Fig. 3d). The average number of PD-L1^+^ cells accumulated around a single TNBC cell in the marginal region was 2.85 (Fig. 3d). The histogram of the distance between PD-L1^+^ cells and TNBC cells revealed that approximately 95% of PD-L1^+^ cells were located within 50 μm of TNBC cells (Fig. 3e).

### Multiplex IF studies of PD-L1

**Fig. 4:**
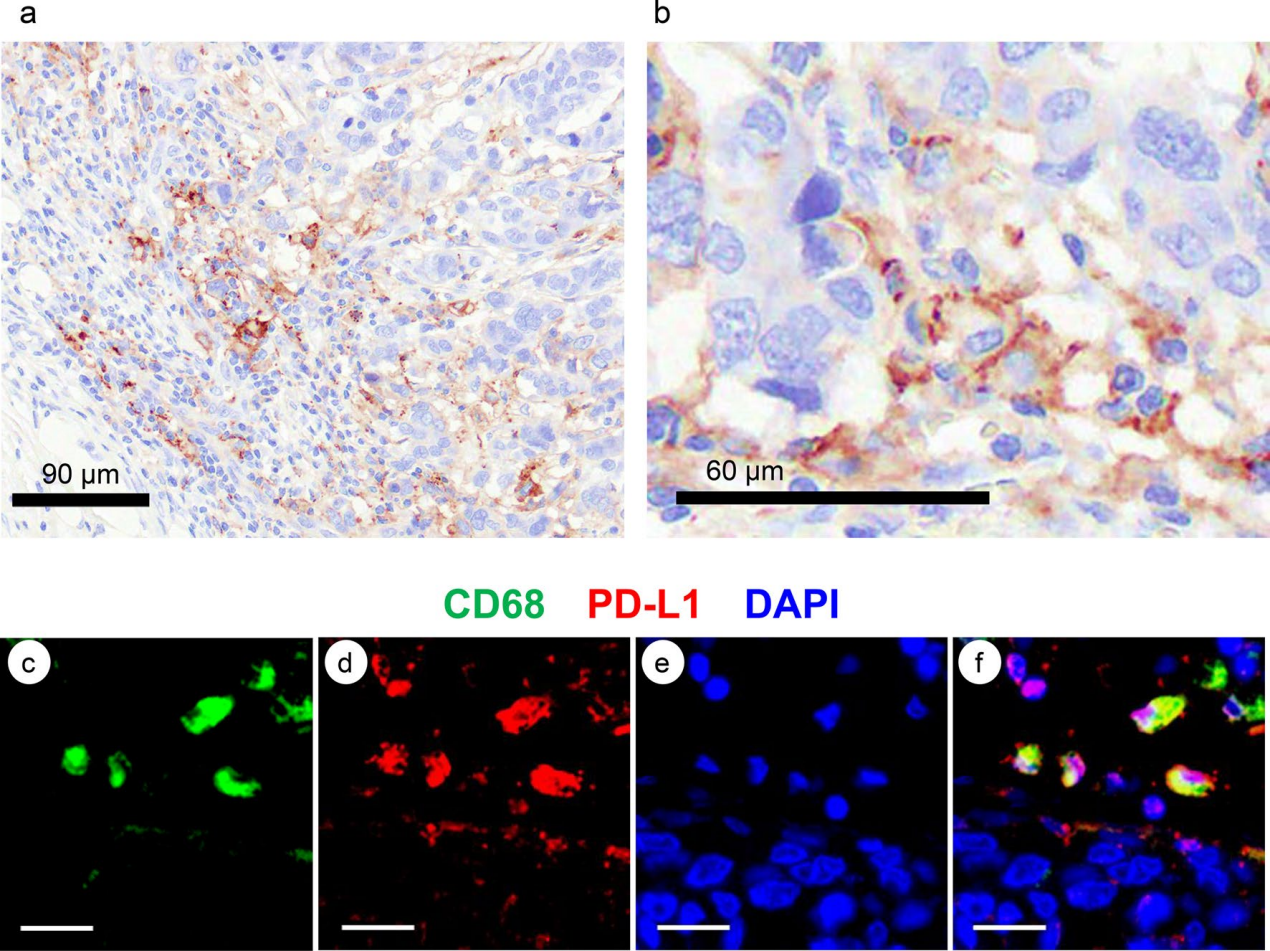
Representative images of PD-L1^+^ cells by multiplex IF studies. **a** PD-L1^+^ stromal cells and PD-L1^−^ TNBC cells. **b** PD-L1^+^ stromal cells at higher magnification. MΦs may be positive for PD-L1. **c** Multiplex IF staining of CD68. CD68^+^ cells were stained with FITC (green). **d** Multiplex IF staining of PD-L1. PD-L1^+^ cells were stained with TRITC (red). **e** The nuclei of the cells were stained with DAPI (blue). **f** Merged image of c, d and e. Bars; 20 μm

We confirmed that stromal cells were positive for PD-L1, while TNBC cells were negative in 30.7% of TNBC cases (Fig. 4a). PD-L1^+^ cells were expected to be identified as MΦs at higher magnification (Fig. 4b). By multiplex IF staining, CD68^+^ cells, PD-L1^+^ cells, and nuclei of the cells were stained with FITC (green) (Fig. 4c), TRITC (red) (Fig. 4d), and DAPI (blue) (Fig. 4e), respectively. The merged image (yellow) indicated that most CD68^+^ cells were also positive for PD-L1 in the marginal region (Fig. 4f).

### Diffuse infiltration of CD68^+^ cells

**Fig. 5:**
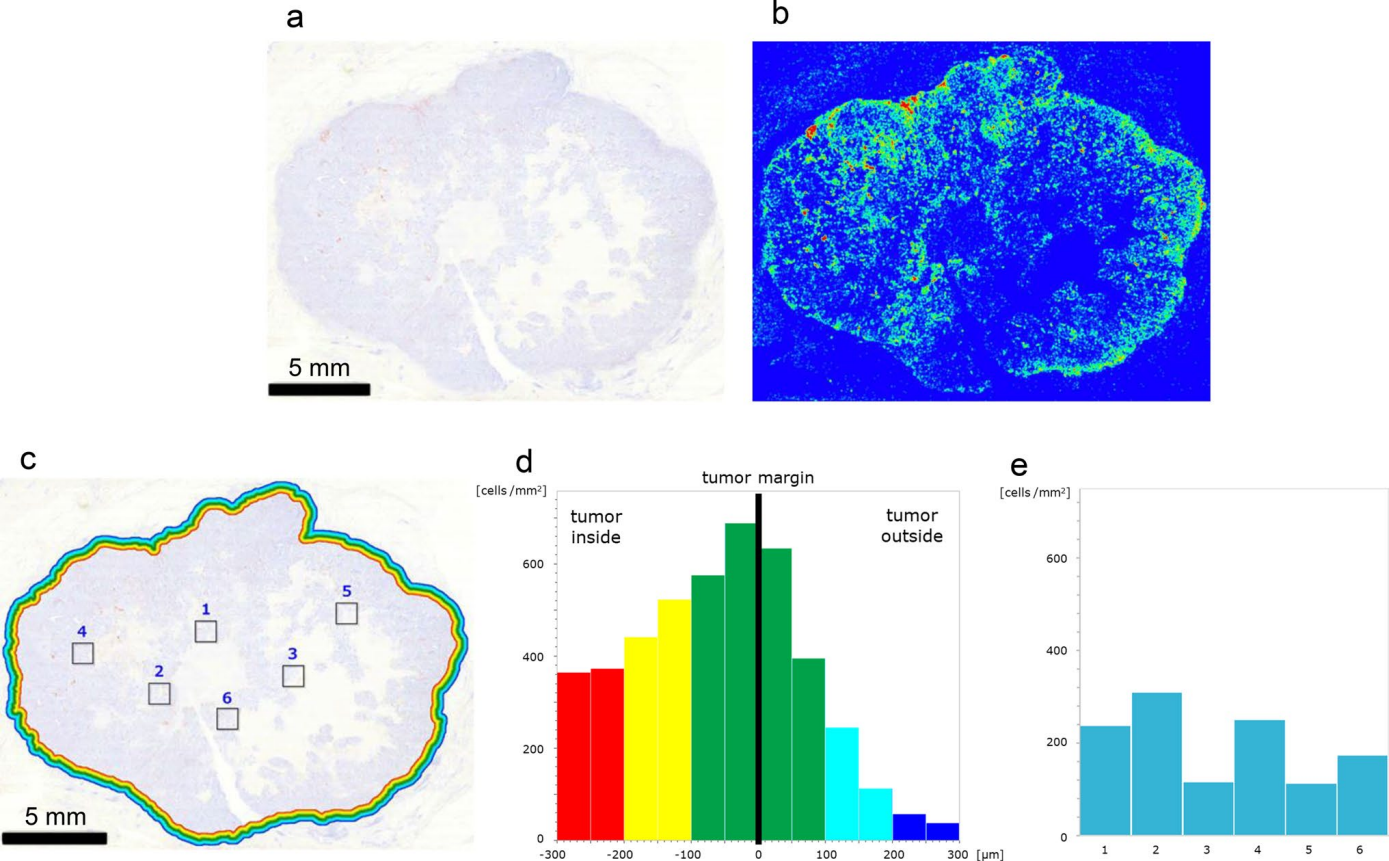
Diffuse infiltration of CD68^+^ cells. **a** CD68^+^ cells were observed in the marginal region and the core region. **b** The heatmap image generated by HALO confirmed the diffuse infiltration of CD68^+^ cells. **c** Quantitative analyses of the density of CD68^+^ cells in the marginal region and core region. **d** The density of CD68^+^ cells near the tumor margin. The marginal region was divided into twelve areas. **e** Density of CD68^+^ cells in the core region

The IHC study of CD68 showed diffuse infiltration of CD68^+^ cells in the TNBC specimen (Fig. 5a). The heatmap image generated by HALO revealed the high-density areas of CD68 cells in the marginal region and the core region (Fig. 5b). The marginal region was divided into twelve areas from 300 μm inside to 300 μm outside the tumor margin, and the density of CD68^+^ cells was examined in each area (Fig. 5c). Histogram analysis revealed that the density of CD68^+^ cells gradually increased toward the tumor margin. The highest density was 2482 per mm^2^ in the column of – 50–0 μm from the tumor margin, and the density gradually decreased (Fig. 5d). To examine the density of CD68^+^ cells in the core region, we randomly chose six square areas (Fig. 5c) and found that the density of CD68^+^ cells in these areas was between 115 and 308 cells per mm^2^ (Fig. 5e).

### Accumulation of CD8^+^ cells and proximity of CD8^+^ cells to PD-L1^+^ cells

**Fig. 6:**
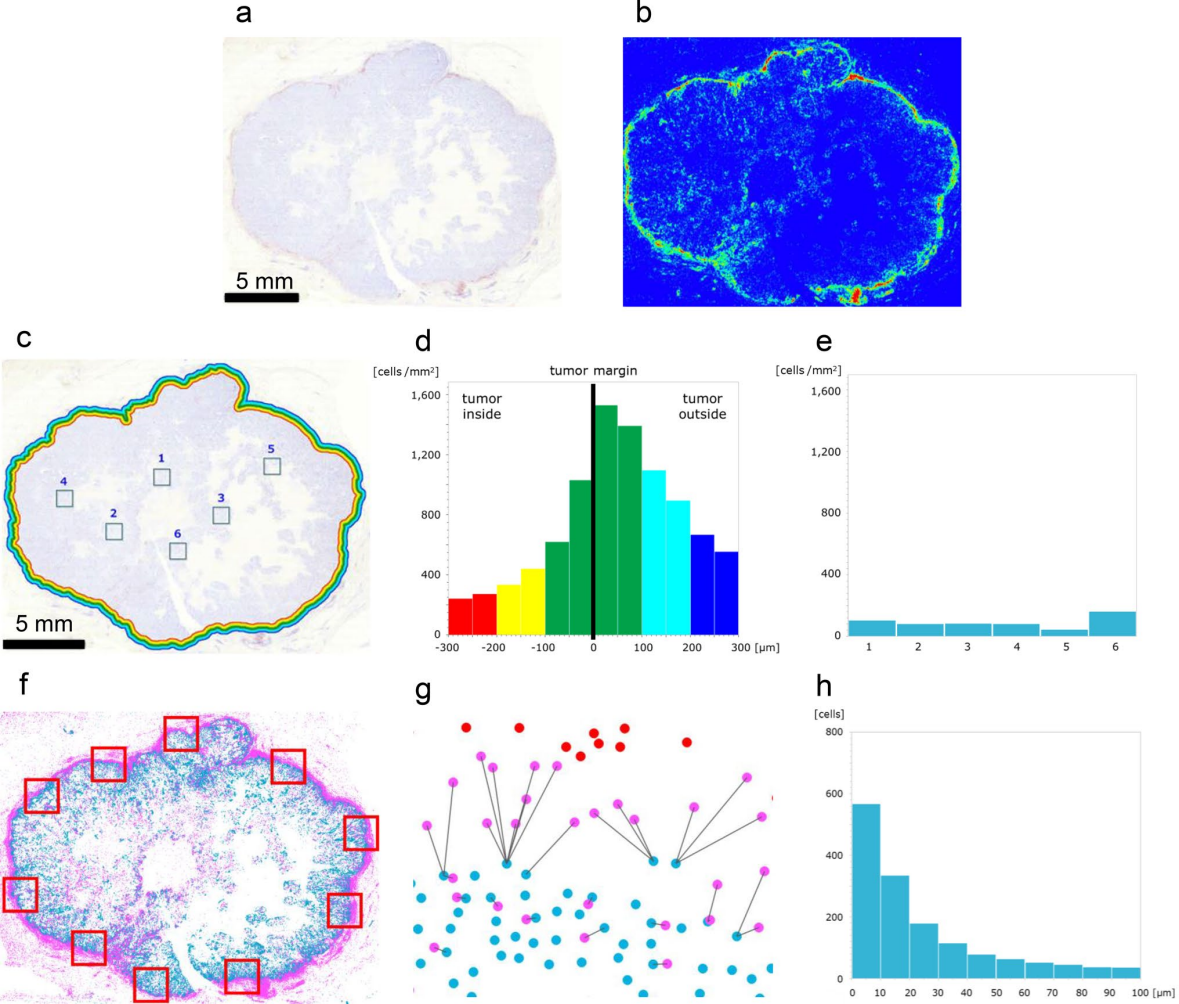
Accumulation of CD8^+^ cells and proximity of CD8^+^ cells to PD-L1^+^ cells. **a** CD8^+^ cells were observed in the marginal region. **b** The heatmap image generated by HALO confirmed the accumulation of CD8^+^ cells in the marginal region. **c** Quantitative analyses of the density of CD8^+^ cells in the marginal region and core region. **d** Density of CD8^+^ cells near the tumor margin. The marginal region was divided into twelve areas. **e** Density of CD8^+^ cells in the core region. **f** Synchronized chart of CD8^+^ cells (purple dots) and PD-L1 cells (light blue dots). In ten square areas in the marginal region, the distance between CD8^+^ cells and PD-L1^+^ cells was examined. **g** Shortest lines from CD8^+^ cells to PD-L1^+^ cells; CD8^+^ cells within 50 μm from PD-L1^+^ cells (purple dots), CD8^+^ cells more than 50 μm away (red dots). **h** Histogram analysis of the distance between CD8^+^ cells and PD-L1^+^cells. Approximately 85% of CD8^+^ cells were located within 50 μm of PD-L1^+^ cells

IHC study of CD8 revealed that CD8^+^ cells accumulated in the marginal region (Fig. 6a). The heatmap image generated by HALO revealed the high density of CD8^+^ cells in the marginal region (Fig. 6b). The marginal region was divided into twelve areas from 300 μm inside to 300 μm outside the tumor margin, and the density of CD8^+^ cells was examined in each area (Fig. 6c). Histogram analysis revealed that the density of CD8^+^ cells gradually increased toward the tumor margin. The highest density was 6039 per mm^2^ in the column of 0–50 μm outside from the tumor margin, and then the density gradually decreased (Fig. 6d). To examine the density of CD8^+^ cells in the core region, we randomly chose six square areas (Fig. 6c), and the density of CD8^+^ cells in these areas was between 43 and 161 per mm^2^ (Fig. 6e). HALO allocated CD8^+^ cells to purple dots in the grid chart and PD-L1^+^ cells to light blue dots. Purple dots and light-blue dots were synchronized in one chart (Fig. 6f). We randomly chose ten square areas in the marginal region (Fig. 6f). In each area, HALO was used to draw the shortest line between CD8^+^ cells and PD-L1^+^ cells (Fig. 6g). CD8^+^ cells within 50 μm of PD-L1^+^ cells were allocated to purple dots, and cells more than 50 μm away from PD-L1^+^ cells were allocated to red dots (Fig. 6g). We counted the number of CD8^+^ cells per single PD-L1^+^ cell in each area (Fig. 6g). The average number of CD8^+^ cells accumulated around a single PD-L1^+^ cell in the marginal region was 2.54 (Fig. 6g). The histogram of the distance between CD8^+^ cells and PD-L1^+^ cells revealed that approximately 85% of CD8^+^ cells were located within 50 μm of PD-L1^+^ cells (Fig. 6h).

### Multiplex IF studies of CCL2 and p-STAT3

**Fig. 7:**
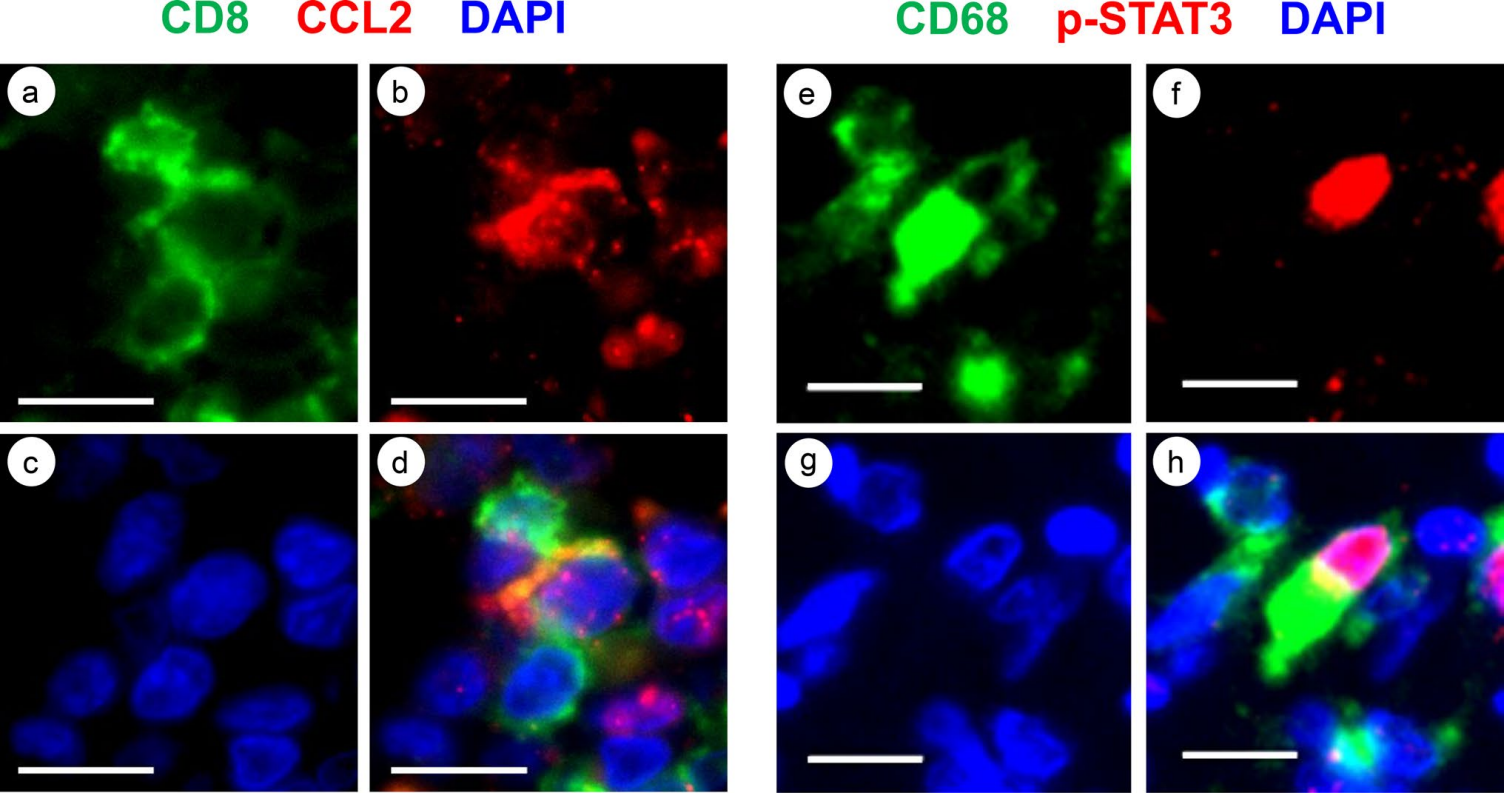
Representative images of CCL2^+^ cells and p-STAT3^+^ cells by multiplex IF studies. **a** Multiplex IF staining of CD8. CD8^+^ cells were stained with FITC (green). **b** Multiplex IF staining of CCL2. CCL2^+^ cells were stained with TRITC (red). **c** The nuclei of the cells were stained with DAPI (blue). **d** Merged image of a, b and c. Bars; 10 μm. **e** Multiplex IF staining of CD68. CD68^+^ cells were stained with FITC (green). **f** Multiplex IF staining of p-STAT3. p-STAT3^+^ cells were stained with TRITC (red). **g** The nuclei of the cells were stained with DAPI (blue). **h** Merged image of e, f and g. Bars; 10 μm

The JAK/STAT and PI3K/AKT/NF-κB signaling pathways activated by inflammatory cytokines have been shown to increase the expression of PD-L1 [25, 26], and C–C motif chemokine ligand 2 (CCL2) has been shown to activate the JAK/STAT and PI3K/AKT/NF-κB signalings [27]. CCL2 was successfully stained in the TNBC specimens by multiplex IF. CD8^+^ cells, CCL2^+^ cells, and the nuclei of the cells were stained with FITC (green) (Fig. 7a), TRITC (red) (Fig. 7b), and DAPI (blue) (Fig. 7c), respectively. The merged image (yellow) indicated that CD8^+^ cells were also positive for CCL2 (Fig. 7d). We also examined multiplex IF studies of CD68 and phospho-signal transducer and activator of transcription 3 (p-STAT3), which was the downstream molecule phosphorylated through JAK/STAT signaling by CCL2. CD68^+^ cells, p-STAT3^+^ cells, and the nuclei of the cells were stained with FITC (green) (Fig. 7e), TRITC (red) (Fig. 7f), and DAPI (blue) (Fig. 7g), respectively. The merged image indicated that CD68^+^ cells were also positive for p-STAT3 (Fig. 7h). We tried to examine the multiplex IF studies of CD68 and phospho-nuclear factor of kappa light polypeptide gene enhancer in B cells inhibitor-alpha (p-IκBα), which was the downstream molecule phosphorylated by PI3K/AKT/NF-κB signaling. However, we could not observe the positivity of p-IκBα in CD68^+^ cells in our examination. In the specimen in which PD-L1^+^ TNBC cells were observed, we examined multiplex IF studies of CD68 and CCL2. We found that CD68^+^ cells were also positive for CCL2 (Supplemental Fig. S2a-d). We confirmed that TNBC cells were positive for PD-L1 in the same specimen (Supplement Fig. S2e). TNBC cells have been demonstrated to express CCR2, which is the receptor of CCL2 [28]. We also found that TNBC cells were positive for p-STAT3 (Supplement Fig. S2f).

## Discussion

In TNBC specimens, the expression of PD-L1 has been shown to be increased in stromal cells and cancer cells [9, 10]. In this study, we observed PD-L1^+^ stromal cells and PD-L1^−^ TNBC cells in 30.7% of our cases and PD-L1^+^ stromal cells and PD-L1^+^ TNBC cells in 18.8% of our cases.

PD-L1^+^ cells have been demonstrated to be localized at the tumor stroma in the marginal region of TNBC specimens [23, 24] and melanoma specimens [21]. We found that PD-L1^+^ cells were localized in the marginal region of TNBC specimen in which PD-L1^+^ stromal cells and PD-L1^−^ TNBC cells were observed. Therefore, we hypothesized that the tumor-stromal interaction in the marginal region would be involved in the induction of PD-L1^+^ cells.

We found that approximately three PD-L1^+^ cells on average accumulated around a single TNBC cell in the marginal region and found that approximately 95% of PD-L1^+^ cells were located within 50 μm of TNBC cells. These results indicate that PD-L1^+^ cells would interact with TNBC cells in the marginal region. These cells would interact at a distance equivalent to the diameter of approximately two and a half MΦs because human MΦs are approximately 20 μm in diameter [29]. Because PD-L1^+^ cells have been demonstrated to suppress antitumor immunity by T cells [6, 7], PD-L1^+^ cells accumulated in the marginal region would protect TNBC cells from attack by CD8^+^ T cells in our study. Because the interaction between MΦs and TNBC cells has been demonstrated to facilitate the induction of TNBC cells with an aggressive phenotype [30], the interaction between PD-L1^+^ MΦs and TNBC cells would also facilitate the growth of TNBC under antitumor immunity.

PD-L1 has been reported to be expressed in MΦs in TNBC [10], non-small cell lung carcinoma [31], and hepatocellular carcinoma [32]. Because we found that PD-L1^+^ cells were CD68^+^ MΦs, our data were consistent with previous reports.

MΦs have been shown to be localized in the marginal region and the core region of TNBC specimens [33, 34]. Although we confirmed the diffuse infiltration of CD68^+^ MΦs in TNBC specimens, we found that PD-L1^+^ MΦs accumulated in the marginal region. Therefore, we hypothesized that the expression of PD-L1 would be induced in MΦs by the interaction with stromal cells at the marginal microenvironment through the cytokines.

Generally, CD8^+^ T cells are the main immune cells at the tumor microenvironment [35]. The following two roles of CD8^+^ T cells in cancer tissues have been documented. One is that CD8^+^ T cells attack cancer cells [22]. The other is that CD8^+^ T cells induce PD-L1 expression in interacting cells [36]. CD8^+^ T cells have been reported to induce PD-L1^+^ cells in melanomas [36]. The induction of PD-L1^+^ stromal cells has been demonstrated when lymphocytes infiltrate into the marginal region of TNBC specimens [23]. If CD8^+^ T cells would attack cancer cells, they would be expected to infiltrate into the core region of tumor specimen [23, 24]. We found that CD8^+^ T cells accumulated in the marginal region of TNBC specimen in which PD-L1^+^ stromal cells and PD-L1^−^ TNBC cells were observed. Therefore, the major role of CD8^+^ T cells would be the induction of PD-L1^+^ cells in our cases, rather than attacking TNBC cells. We also found that approximately three CD8^+^ cells accumulated around a single PD-L1^+^ cell and found that approximately 85% of CD8^+^ cells were located within 50 μm of PD-L1^+^ cells, so CD8^+^ cells would interact with PD-L1^+^ cells in the marginal region. These findings indicate that CD8^+^ T cells would be involved in the induction of PD-L1^+^ MΦs at the marginal microenvironment of TNBC specimens. We also expected that CD8^+^ T cells would induce PD-L1 expression in MΦs through the cytokines.

Inflammatory cytokines such as interferon (IFN)-γ, tumor necrosis factor (TNF)-α, interleukin (IL)-1β, IL-6, and IL-10 have been shown to be involved in the regulation of PD-L1 expression [6, 7]. The JAK/STAT and PI3K/AKT/NF-κB signaling pathways activated by inflammatory cytokines such as IFN-γ and interleukins have been shown to increase the expression of PD-L1 in vitro and in vivo [25, 26]. CCL2 has been demonstrated to play major roles in the recruitment of inflammatory monocytes as well as their differentiation into MΦs [37, 38]. CCL2 and its receptor CCR2 have also been shown to activate the JAK/STAT and PI3K/AKT/NF-κB signaling in vitro and in vivo [27]. We expected that CD8^+^ T cells may induce the expression of PD-L1 in MΦs through the activation of the JAK/STAT and PI3K/AKT/NF-κB signaling by CCL2. We found that CD8^+^ T cells produced CCL2 and that CD68^+^ cells were positive for p-STAT3 in the marginal region of TNBC specimen. Our results suggest that CCL2 derived from CD8^+^ T cells would induce the expression of PD-L1 in MΦs through the activation of JAK/STAT signaling at the marginal microenvironment. Although STAT3 is demonstrated to be a transducer of other cytokines, CCR2 knockout study has shown to reduce PD-L1 expression in cancer cells [39].

It has been shown that the activation of PI3K/AKT/NF-κB signaling could induce both PD-L1 and CCL2 expression independently, and CCL2 might cause the resistance to PD-1/PD-L1 inhibitors in TNBC [40]. Therefore, it is possible that the expression of both PD-L1 and CCL2 in MΦs results from the activation of PI3K/AKT/NF-κB signaling by other inflammatory cytokines produced by CD8^+^ T cells. However, we could not demonstrate the positive findings by multiplex IF studies of p-IκBα and CD68. MΦs have been demonstrated to promote cancer progression through CCL2 [27]. We also found that CD68^+^ MΦs produced CCL2 and that TNBC cells were positive for p-STAT3 in the specimen in which PD-L1^+^ TNBC cells were observed. These findings suggest that CCL2 derived from CD68^+^ MΦs might induce the expression of PD-L1 in TNBC cells. The molecular mechanisms of the induction of PD-L1 expression by CCL2 should be elucidated in future studies.

In conclusion, we found that PD-L1^+^ MΦs would be induced by interaction with CD8^+^ T cells through CCL2 at the marginal microenvironment of TNBC specimens. The interaction between PD-L1^+^ MΦs and TNBC cells would facilitate the growth of TNBC under antitumor immunity. These interactions would be potential targets for restoring antitumor immunity and suppressing TNBC progression.

### Supplementary Information

Below is the link to the electronic supplementary material.

Supplementary file 1: (PDF 554 KB)
