# Supplementary material for: Histological spatial analysis on the induction of PD-L1+ macrophages by CD8+ T cells at the marginal microenvironment of triple-negative breast cancer
Source: Breast Cancer. 2023 Oct 4;30(6):1094–104. doi: 10.1007/s12282-023-01507-9 (PMC10587303; doi:10.1007/s12282-023-01507-9)
Supplement: Supplementary file 1 — (PDF 554 KB) [file 12282_2023_1507_MOESM1_ESM.pdf]

Supplemental Information

Histological spatial analysis on the induction of PD-L1^{+} macrophages by CD8^{+} T cells at the marginal microenvironment of triple-negative breast cancer

Kazushi Suzuki[1*], Rintaro Ohe[1], Takanobu Kabasawa[1], Takumi Kitaoka[1], Masaaki Kawai[2], Fuyuhiko Motoi[2], and Mitsuru Futakuchi[1]

[1] Department of Pathology, Yamagata University Faculty of Medicine, Yamagata, Japan

[2] Department of Surgery 1, Yamagata University Faculty of Medicine, Yamagata, Japan

***Corresponding author:** Kazushi Suzuki, M.D., Department of Pathology, Yamagata University Faculty of Medicine, 2-2-2 Iida-Nishi, Yamagata 990-9585, Japan.

E-mail address: kazu.suzuki@med.id.yamagata-u.ac.jp

Telephone number: +81(0)-23-628-5238

Facsimile number: +81(0)-23-628-5240

ORCID: 0000-0003-4953-8656

Supplemental Table

Supplemental table S1. The clinicopathological characteristics for TNBC patients (N=101)

	PD-L1 positivity (stromal cells/TNBC cells) [n/N(%)]		
	(-/-)	(+/-)	(+/+)
	(N=51)	(N=31)	(N=19)
Age (median [range]) (years)	66 [36-91]	56.5 [28-90]	62 [35-79]
Pathological stage			
Stage I	19/51 (37.3)	14/31 (45.2)	5/19 (26.3)
Stage II	26/51 (51.0)	16/31 (51.6)	13/19 (68.4)
Stage III	6/51 (11.8)	1/31 (3.2)	1/19 (5.3)
Nuclear grade			
Grade 1	17/51 (33.3)	0/31 (0.0)	2/19 (10.5)
Grade 2	17/51 (33.3)	5/31 (16.1)	5/19 (26.3)
Grade 3	17/51 (33.3)	26/31 (83.9)	12/19 (63.2)
BRCA1/2 gene mutations (n)			
BRCA1	0	1	2
BRCA2	0	0	0

Supplemental Figures

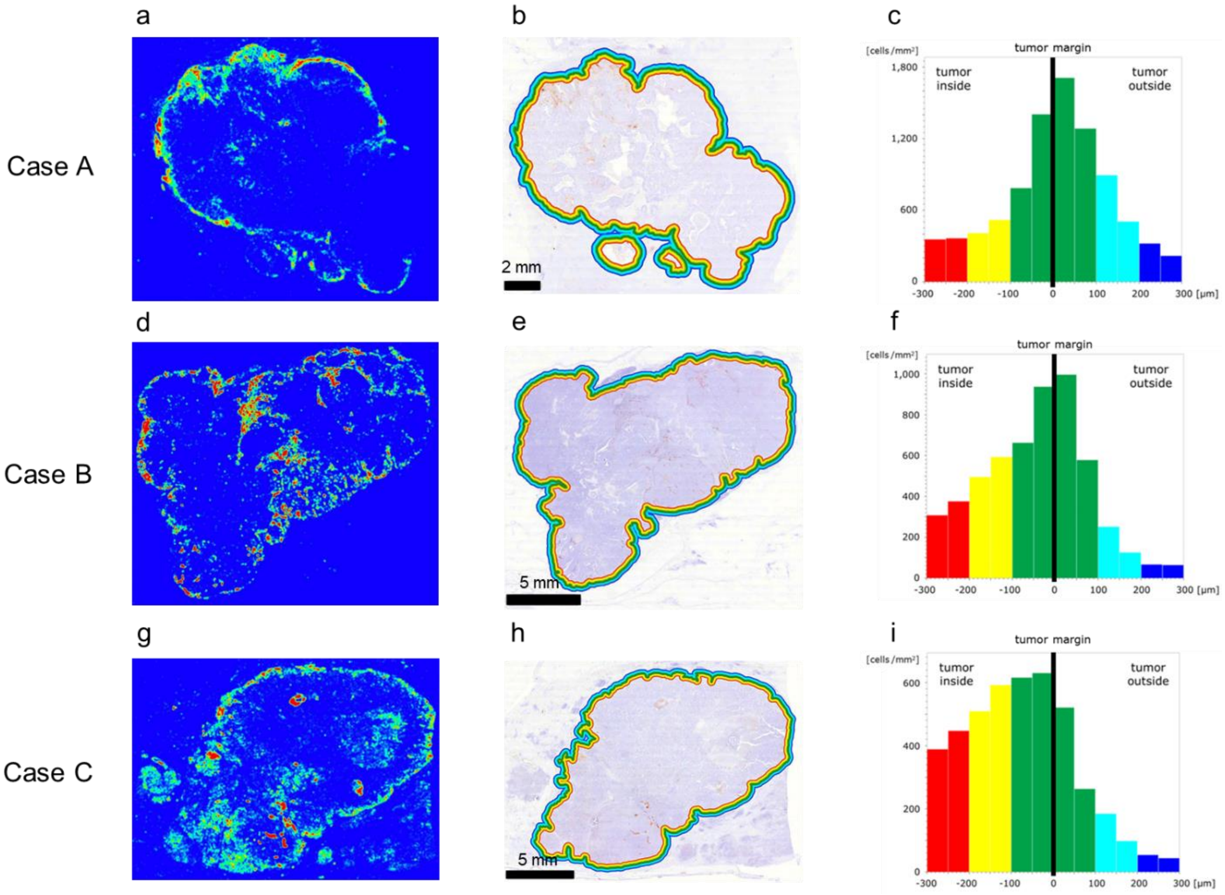

Supplemental Figure S1.

a The heatmap image confirmed the accumulation of PD-L1$^+$ cells in the marginal region (Case A). **b** Quantitative analysis of the density of PD-L1$^+$ cells in the marginal region (Case A). **c** The density of PD-L1$^+$ cells near the tumor margin (Case A). **d** The heatmap image confirmed the accumulation of PD-L1$^+$ cells in the marginal region (Case B). **e** Quantitative analysis of the density of PD-L1$^+$ cells in the marginal region (Case B). **f** The density of PD-L1$^+$ cells near the tumor margin (Case B). **g** The heatmap image confirmed the accumulation of PD-L1$^+$ cells in the marginal region (Case C). **h** Quantitative analysis of the density of PD-L1$^+$ cells in the marginal region (Case C). **i** The density of PD-L1$^+$ cells near the tumor margin (Case C).

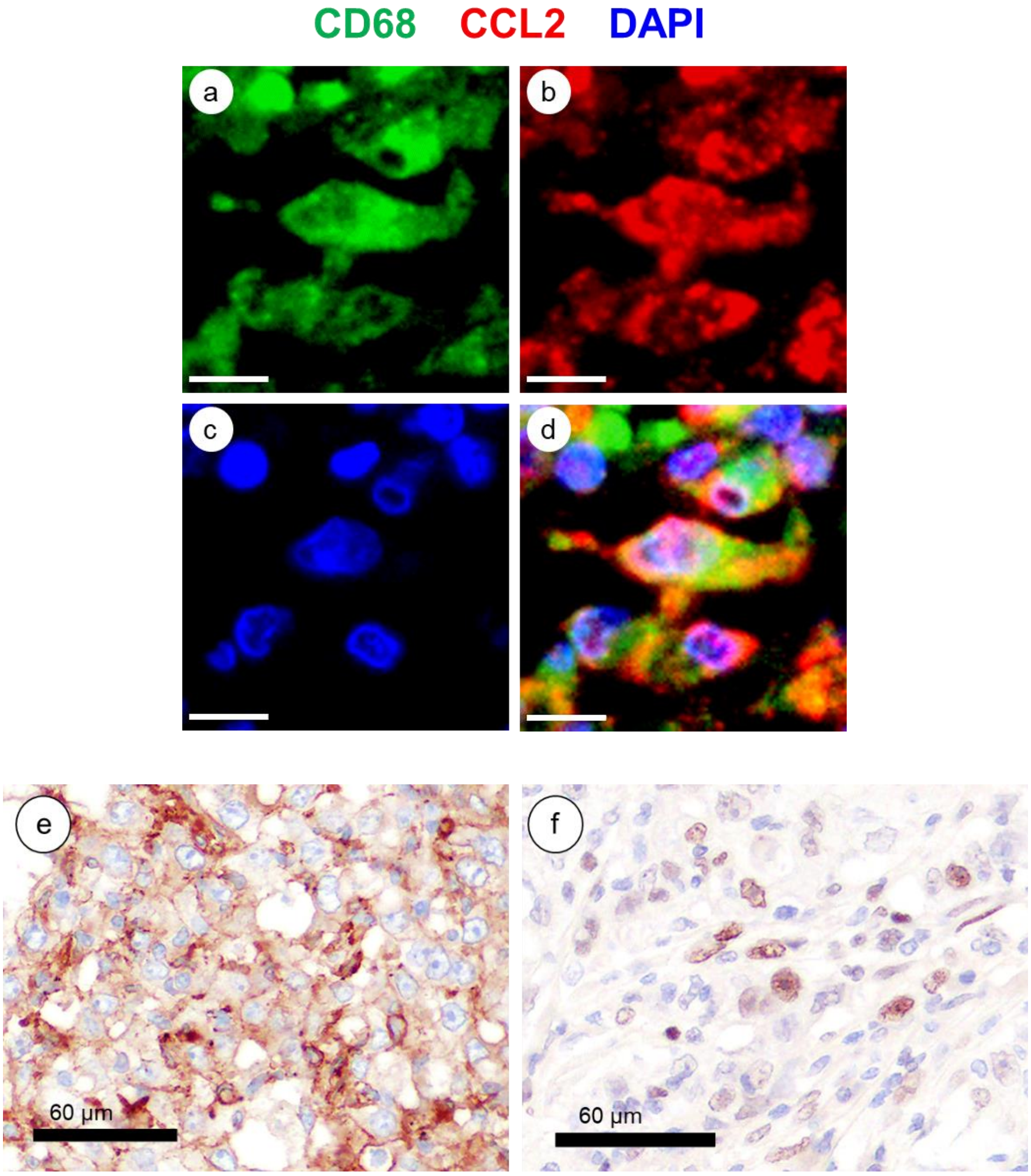

Supplemental Figure S2.

a Multiplex IF staining of CD68. CD68^{+} cells were stained with FITC (green). **b** Multiplex IF staining of CCL2. CCL2^{+} cells were stained with TRITC (red). **c** The nuclei of the cells were stained with DAPI (blue). **d** Merged image of a, b and c. Bars; 10 µm. **e** PD-L1^{+} TNBC cells were observed. **f** TNBC cells were also positive for p-STAT3.
